# Electrolysis without electrodes: thermodynamic and kinetic insights into decoupled alkaline water electrolysis

**DOI:** 10.1039/d6fd00117c

**Published:** 2026-08-04

**Authors:** Mark Potter, Luís Pinho, Kathryn E. Toghill

**Affiliations:** a Department of Chemistry, Lancaster University Lancaster LA1 5UB UK k.toghill@lancaster.ac.uk

## Abstract

Conventional water electrolysis is fundamentally limited by the kinetic constraints of coupled electrode reactions, particularly the oxygen evolution reaction, which necessitate substantial overpotentials despite decades of catalyst development. Recently, spatially and temporally decoupled electrolysis has emerged as a new paradigm in which electrochemical charge storage and catalytic fuel production are separated. This decoupling enables catalysis beyond the confines of the electrode surface and fundamentally alters how electrons are delivered to the catalyst. Here, we investigate redox-mediated hydrogen evolution in strongly alkaline media, examining the interplay between dissolved redox mediators and heterogeneous hydrogen evolution catalysts. Notably, we demonstrate that separating electron transfer from catalytic hydrogen evolution minimises some of the kinetic penalty associated with the electrode–electrolyte interface, enabling hydrogen evolution at potentials approaching the thermodynamic limit, even on nickel catalysts. Nevertheless, catalyst identity remains critical in determining reaction kinetics. Comparison of platinum and Raney® nickel reveals a prolonged activation period unique to nickel, demonstrating that catalyst surface chemistry continues to govern hydrogen evolution despite the absence of direct electrode-mediated electron transfer. These findings establish decoupled electrolysis as a fundamentally distinct model for investigating the interplay between charge transfer and catalysis, providing new insight into electrocatalytic energy conversion.

## Abstract 2

We investigate redox-mediated hydrogen evolution in strongly alkaline media, examining the interplay between dissolved redox mediators and heterogeneous hydrogen evolution catalysts.

## Graphical abstract

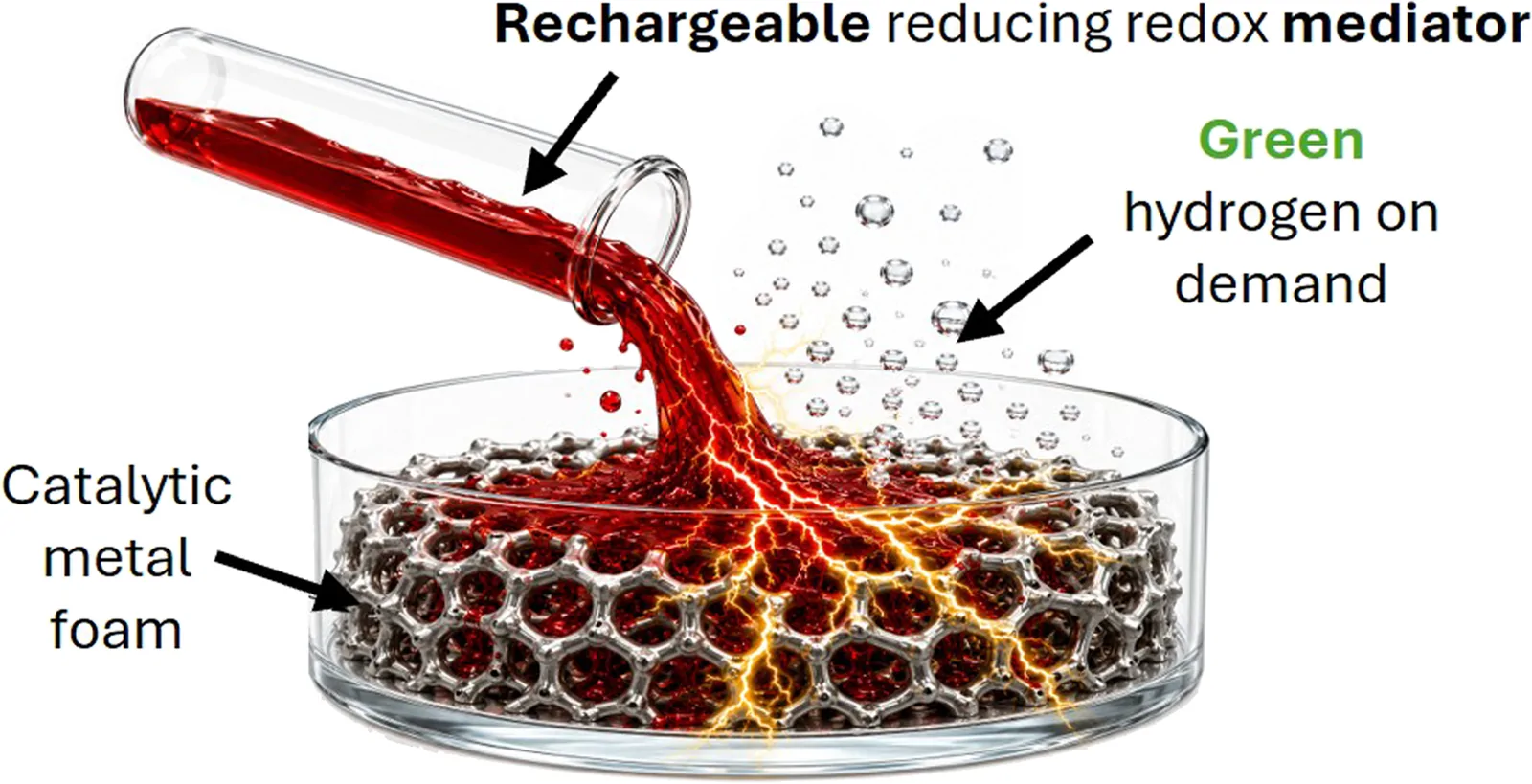

## Introduction

Decoupled electrolysis is an emerging area of research which aims to use the intrinsic potentials of an electrochemically reversible redox mediator species to drive an irreversible chemical process, independently of the electrodes.^1–5^ Here, we consider two distinct categories of decoupled water electrolysis; one in which a soluble redox mediator is electrolysed to a “charged” state and is used to drive a reduction or oxidation reaction, regenerating the “discharged” state in the process (Fig. 1A – dual flow), and a second one where a soluble mediator is used to first support an electrolytic oxygen evolution reaction (OER), and then an electrolytic hydrogen evolution reaction (HER) (Fig. 1C – dual cell). A third, intermediate approach can also be described, where oxygen evolves electrolytically *vs.* the mediator reduction, but the mediator is used in a chemical discharge to generate hydrogen (or *vice versa*) (Fig. 1B – half flow). In all cases, the evolution of hydrogen and oxygen is both spatially and temporally decoupled, so incompatible reaction rates and safety concerns regarding their co-production are avoided. Most of the work in this space has concerned water splitting, but more complex reactions are being reported, such as CO_2_ reduction,^6,7^ aniline synthesis,^8^ biomass oxidation.^9^ and other chemicals.^10^ These are more challenging processes, owing to the introduction of a third reaction species, but demonstrate the versatility of the decoupled electrolysis approach to alternative chemical synthesis.

**Fig. 1:**
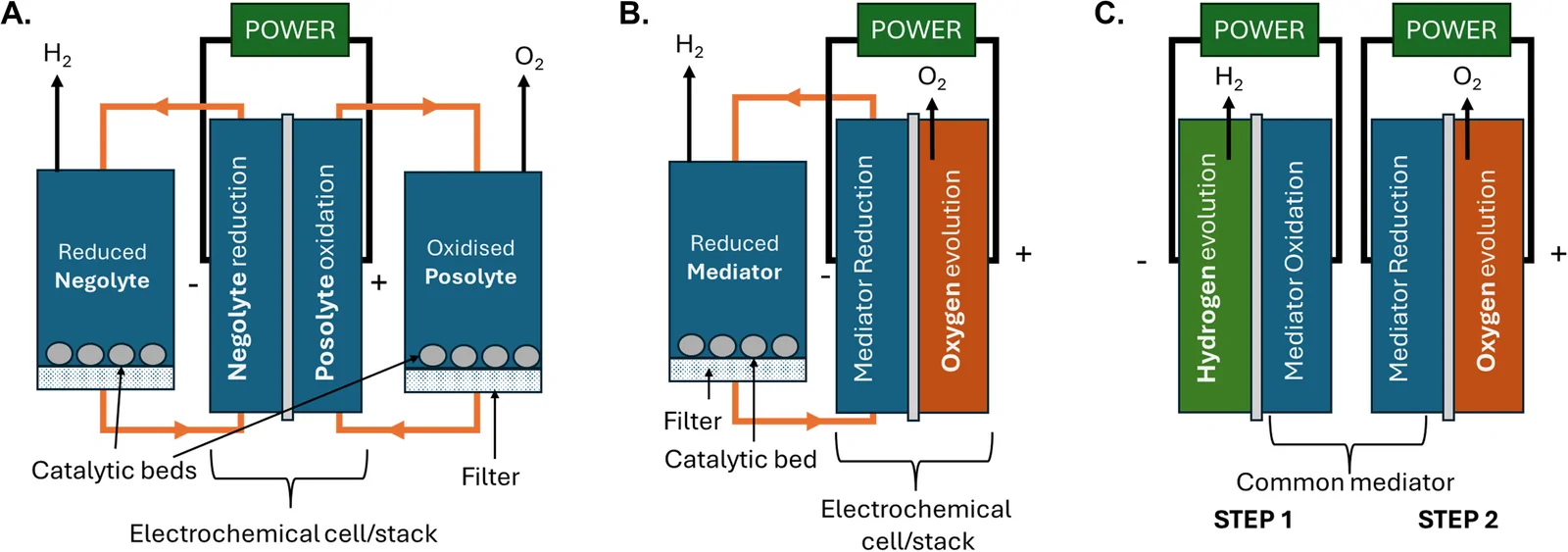
Simple schematics of decoupled electrolysis designs using soluble mediators. (A) The dual flow approach with two distinct positive and negative redox mediators and separate hydrogen and oxygen evolution reactors. (B) The half-flow mediator reduction *vs.* conventional electrolysis and separate hydrogen reactor using reduced mediator. (C) The dual cell approach uses two electrochemical cells with a common mediator, one evolving hydrogen while oxidising the redox mediator, and the second evolving oxygen while reducing the same mediator.

The dual flow (or dual circuit) approach (Fig. 1A) was first reported in 2014 by Girault *et al.*^11^ Using a vanadium/cerium flow battery, the group were able to use the charged states of the battery, V(ii) and Ce(iv), to catalytically drive HER and the OER in separate reactors. Both processes were electrochemically derived but chemically initiated. The approach achieved near 100% conversion efficiency of charged electrolyte to hydrogen and *ca.* 70% conversion efficiency for the oxygen. The approach was explored further, switching the cerium mediator for manganese^12^ and exploring the half-flow approach (Fig. 1B) with vanadium and OER.^13^ At a similar time, Symes and Cronin reported the dual cell (Fig. 1C) approach in which a single polyoxometalate (POM) material was electrochemically reduced *vs.* a half-cell generating oxygen, then oxidised *vs.* a separate half-cell which generated hydrogen.^14^ This latter work has evolved over the years^5^ such that a commercial enterprise to achieve decoupled water electrolysis is underway using the half-flow method (Clyde Hydrogen).^15^

In the decade that has followed more decoupled electrochemical systems have been reported as discussed in a number of reviews.^1,2,5,16^ Alkaline electrolytes comprising phenazines^17,18^ and iron triethanolamine^19^ have been reported for the reduction reaction at pH ≥ 14. The latter work reported quite high faradaic yields *ca.* 70%, which is surprising given the driving force for Fe(TEA) being very close to thermodynamic HER. Other than the application of molybdenum carbide as the catalyst in the inaugural work using acidic vanadium electrolytes,^11^ most reports have relied on platinum to drive HER. With the high propensity of platinum to catalyse water splitting there has been little to no barrier for this process to take place when the redox mediators have had sufficient driving force. The implications of decoupling for electrocatalytic kinetics remain comparatively unexplored.

In recent work, we evaluated the ability of a redox mediator to drive HER in near neutral pH using a range of non-platinum group catalysts. Specifically, we sought to use a high driving force mediator, Cr PDTA, with 600 mV of available overpotential. However, to our surprise the chromium mediated decoupled HER outperformed the best HER catalyst, MoS_2_ when in a electrolyser configuration comprising 1 M KHCO_3_ and excess ferrocyanide. Furthermore, the platinum benchmark catalyst was the worst performing in the bicarbonate buffer when used in a decoupled manner.^20^ It is possible that by separating electrochemical charge accumulation from catalytic product formation, decoupled systems may access reaction pathways and turnover regimes unavailable to conventional interfacial electrolysis.

Here we sought to understand decoupled HER in strongly alkaline conditions using nickel catalysts, evaluating the performance at thermodynamic potentials *vs.* those with higher driving force, but relating to the conventional electrocatalyst performances. Nickel based alkaline HER is a long established and mature technology, but its highest cost is bottom line electricity prices caused in part by high cell overpotentials to drive high yields of hydrogen. By mediating electron delivery and arriving at thermodynamic catalytic potentials, the “cost” of electricity could be substantially lowered. Decoupling separates electrochemical charge accumulation from catalytic bond-forming chemistry, permitting coupled proton/electron transfer processes to proceed in environments distinct from the conventional electrified interface. Thus, we shift from evaluating and improving electrode kinetics to electrochemical system kinetics.

## Methods

### Materials, chemicals and reagents

All chemicals were used as purchased without further purification. 2,5-Dihydroxy-1,4-benzoquinone, iron(iii) chloride hexahydrate (97%), 2-hydroxy-1,4-naphthoquinone (≥96.5%), 3,4-diaminobenzoic acid (97%), Raney® Ni 2800 slurry and platinized Vulcan (Pt-V10, 10% weight loading) were obtained from Merck (Sigma-Aldrich). Iron-2,2-bis(hydroxymethyl)-2,2′,2′-nitrilotriethanol (98%, Bis-Tris), 3-[bis(2-hydroxyethyl)amino]-2-hydroxypropanesulfonic acid (99%, DIPSO), potassium ferrocyanide (99%), nickel powder APS 2.2–3 micron and nickel powder APS 3–7 micron (APS, atmospheric plasma spraying, 99.9%), and acetone were purchased from Thermo Fisher Scientific (Alfa Aesar, Acros Organics). 3,4-Diaminobenzenesulfonic acid (97%) was obtained from BLDpharm. All water used in the experiments was Millipore deionised water with a resistivity of 18 MΩ cm^2^.

### Synthesis

#### Dihydroxyphenazine sulfonate (DHPS)

DHPS was synthesised according to Hollas *et al.*^21^ A round-bottomed flask was filled with 130 mL of water and warming to 105 °C in an oil bath was begun. While warming, solid 2,5-dihydroxy-1,4-benzoquinone (8.278 g, 0.059 mol) was added to the reaction flask. Then, over a 5 min period, solid 3,4-diaminobenzenesulfonic acid (11.106 g, 0.059 mol) was added to the reaction mixture. After refluxing overnight, the reaction mixture was cooled to room temperature, diluted with 150 mL of acetone and then filtered to yield a gold solid, which was washed with water and then acetone. The solid was dried under vacuum for six days to yield the product (17.063 g, 99%).

#### 5-Hydroxy-benzo[*a*]phenazine-sulfonic acid (BHPS)

BHPS was synthesised following procedures outlined by Park *et al.*^22^ 2-Hydroxy-naphthoquinone (300.2 mg, 1.724 mmol) was slowly added to a solution of 3,4-diaminobenzenesulfonic acid (324.5 mg, 1.724 mmol) in glacial acetic acid (50 mL) and stirred overnight at 60 °C. The reaction mixture was poured into water (150 mL) whilst stirring, and the precipitate vacuum-filtered and dried to give a bright orange solid.

#### Iron-2,2-bis(hydroxymethyl)-2,2′,2′-nitrilotriethanol (Fe Bis-Tris)

Fe Bis-Tris was synthesised following procedures outlined by Shin *et al.*^23^ 1.35 g of FeCl_3_·H_2_O were dissolved into 100 mL of deionized water and the mixture was stirred until complete dissolution. 2.09 g of Bis-Tris were added to the mixture and stirred until complete dissolution to give a 1 : 2 Fe : Bis-Tris ratio. Then, 40 g of NaOH were added and left to stir overnight. The volume of the mixture solution was adjusted to 250 mL. All processes were carried out under N_2_ purge.

#### Iron-3-[bis(2-hydroxyethyl)amino]-2-hydroxypropanesulfonic acid (Fe DIPSO)

Fe DIPSO was synthesised following procedures outlined by Shin *et al.*^24^ 1.35 g of FeCl_3_·H_2_O were dissolved into 100 mL of deionized water and the mixture was stirred until complete dissolution. 2.43 g of DIPSO were added to the mixture and stirred until complete dissolution. Then, 40 g of NaOH were added and left to stir overnight. The volume of the mixture solution was adjusted to 250 mL. All processes were carried out under N_2_ purge.

### Electrode preparation

Voltammetry used a conventional three-electrode cell comprising a glassy carbon working electrode, a Hg/HgO reference electrode and platinum wire counter electrode (all from ALS). The working electrode was polished prior to use using 50 nm average particle size alumina powder.

To evaluate the voltammetry of the catalyst candidates, ink suspensions were prepared to uniformly modify the inert glassy carbon electrode. 5 mg of catalyst and 500 µl of deionised water were mixed before ultrasonication for 30 min to give a dispersion of 10 mg mL^−1^. To this, 50 µl of Nafion dispersion (2 wt%) were added and the mixture further sonicated for 5 minutes. A volume of 5 µl of the sonicated inks was drop cast onto the glassy carbon electrode giving approximate coverage of 0.64 mg cm^−2^.

### Electrochemical methods

Electrochemical experiments were performed using an IVIUM n-Stat. Voltammetric characterisation was performed in a standard three-electrode cell using a 3 mm diameter glassy carbon working electrode, Pt coil counter electrode and an Hg/HgO reference electrode. Cyclic voltammetry was performed at a scan rate of 100 mV s^−1^ unless otherwise stated. Linear sweep voltammetry was performed at a scan rate of 5 mV s^−1^ with no stirring. The overpotential values used for the HER were corrected to the reversible hydrogen electrode (RHE) according to the following equation:*E vs.* RHE (V) = *E vs.* Hg/HgO (4 M NaOH) + 0.071 + 0.059 × pH (pH = 14.6)where *E vs.* Hg/HgO is the potential measured in the experiment, 0.071 V is the formal potential of the Hg/HgO reference *vs.* SHE in 4 M NaOH, and a pH of 14.6 is estimated for the 4 M NaOH electrolyte.

Mediator charging was conducted galvanostatically in a custom flow cell with an electrode surface area of 16 cm^2^, utilising GFD 4.6 SIGRACELL graphite felt electrodes which had been heat-treated for 4 hours at 500 °C under air to improve hydrophilicity, and a Fumapem® FKE-30 cation exchange membrane (Fumatech GmbH). The redox mediator solution was charged in the custom flow cell against an excess of K_4_[Fe(CN)_6_] under an inert N_2_ atmosphere. Once charged, the solution was transferred to a nitrogen glovebox (Saffron Scientific Ltd).

### Catalyst testing

To facilitate the measurement of mass of a solid instead of a water suspension, RN2800 water suspension was dried overnight under vacuum to obtain dry RN2800. All other catalysts were used as supplied. Batch decoupled reduction reactions were performed in septum-sealed 20 mL gas chromatography vials. 5 mg of catalyst powders were placed in uncapped septum-sealed flasks before being introduced in a glovebox so that HER could develop in inert conditions. 5 mL of the charged mediator were introduced in the flasks for HER to occur. They were then capped and left to react without stirring until completeness. The reaction was considered complete when the colour of the mediator solution returned to its discharged state.

### Gas quantification and product analysis

Gaseous products were analysed by gas chromatography (GC). GC was performed utilising a Shimadzu 2030 equipped with a thermal conductivity detector (TCD) and a ResTek Shin Carbon ST 80/100 column. A calibration curve was obtained for H_2_ in the 0–300 000 ppm range, by use of a 30% H_2_ (N_2_ balance) calibration gas supplied by BOC Ltd, diluted to achieve a range of concentrations (Fig. S1). Samples were extracted from the headspace of septum-sealed flasks and introduced *via* a gas tight syringe (volume 1 mL VICI® precision Sampling Ltd), in the split/splitless inlet of the gas chromatograph.

### Kinetic evaluation

To calibrate the response of the mediator in the presence of the catalyst, a series of intermediate SOC mixtures were prepared by combining fully oxidised and fully reduced (by bulk electrolysis) solutions under inert N_2_ atmosphere. The potentials of the resulting solutions were measured on a glassy carbon working electrode against a Ag/AgCl leakless double junction reference electrode (Innovative Instruments), and the corresponding potential *vs.* SOC plots fit well with the theoretical values.

The kinetic performance of DHPS mediated catalysis was explored using dry RN2800 as a HER catalyst. A solution of 10 mM DHPS in 4 M NaOH was charged in the flow cell at 100 mA. The charged dark orange solution was kept under nitrogen (N_2_) for further use. Aqueous dispersions of RN2800 were prepared and saturated with N_2_. A 100 mL electrochemical cell with glassy carbon working electrode and a leak-free Ag/AgCl reference electrode (Innovative Instruments Ltd) was assembled. To this, 50 mL of mediator were added under magnetic stirring (500 rpm with a magnetic stirrer bar) for the establishment of the initial values of open circuit potential. 5 mL of catalyst dispersion was then added after 2–5 minutes of measurement. Open circuit chronopotentiometry (OCCP) measurements were then taken continuously until full discharge. The glassy carbon electrode was polished between experiments, and the electrochemical cells were cleaned in aqua regia solution to ensure complete removal of the catalyst. To quantify hydrogen production in time, 1 mL of the headspace of the electrochemical cell was sampled at regular time intervals and introduced *via* a gas tight syringe (volume 1 mL VICI® Precision Sampling Ltd) in the split/splitless inlet of the gas chromatograph and quantified against the previously referred calibration curve. All experiments were conducted at least 2 times to ensure reproducibility. As a maximum of 12 mL of gas will be produced over the course of the experiments, and the volume of the headspace is large (110 mL), the effects on pressure of taking many 1 mL aliquots was considered to be negligible.

## Results and discussion

### Electrochemical characterisation of mediators and catalysts

This work sought to evaluate the mediated decoupled hydrogen evolution reaction (deHER) in strongly alkaline media catalysed by nickel materials to directly compare with conventional alkaline electrolysers. Specifically, we sought to understand the relationship of mediator driving force, proton coupled discharge and catalyst ability. The mediators of interest were selected from existing flow battery literature with the key trait being that they are comprised solely of earth abundant elements (no critical raw materials as defined by the European Union). These include: iron complexes of alkanolamines such as triethanolamine (TEA), 3-[bis(2-hydroxyethyl)amino]-2-hydroxypropanesulfonic acid (DIPSO), and 2,2-bis(hydroxymethyl)-2,2′,2′-nitrilotriethanol (Bis-Tris), as well as phenazine derivatives such as 7,8-dihydroxyphenazine-2-sulfonic acid (DHPS) and benzo[*a*]phenazin-5-ol methanesulfonate (BHPS). Their chemical structures are given in Fig. 2. The reversible electrochemical oxidation and reduction of the iron derivatives are not proton coupled reactions and provide one mole of electrons per mole of mediator. The phenazines undergo two-electron-two-proton coupled reduction and oxidation.

**Fig. 2:**
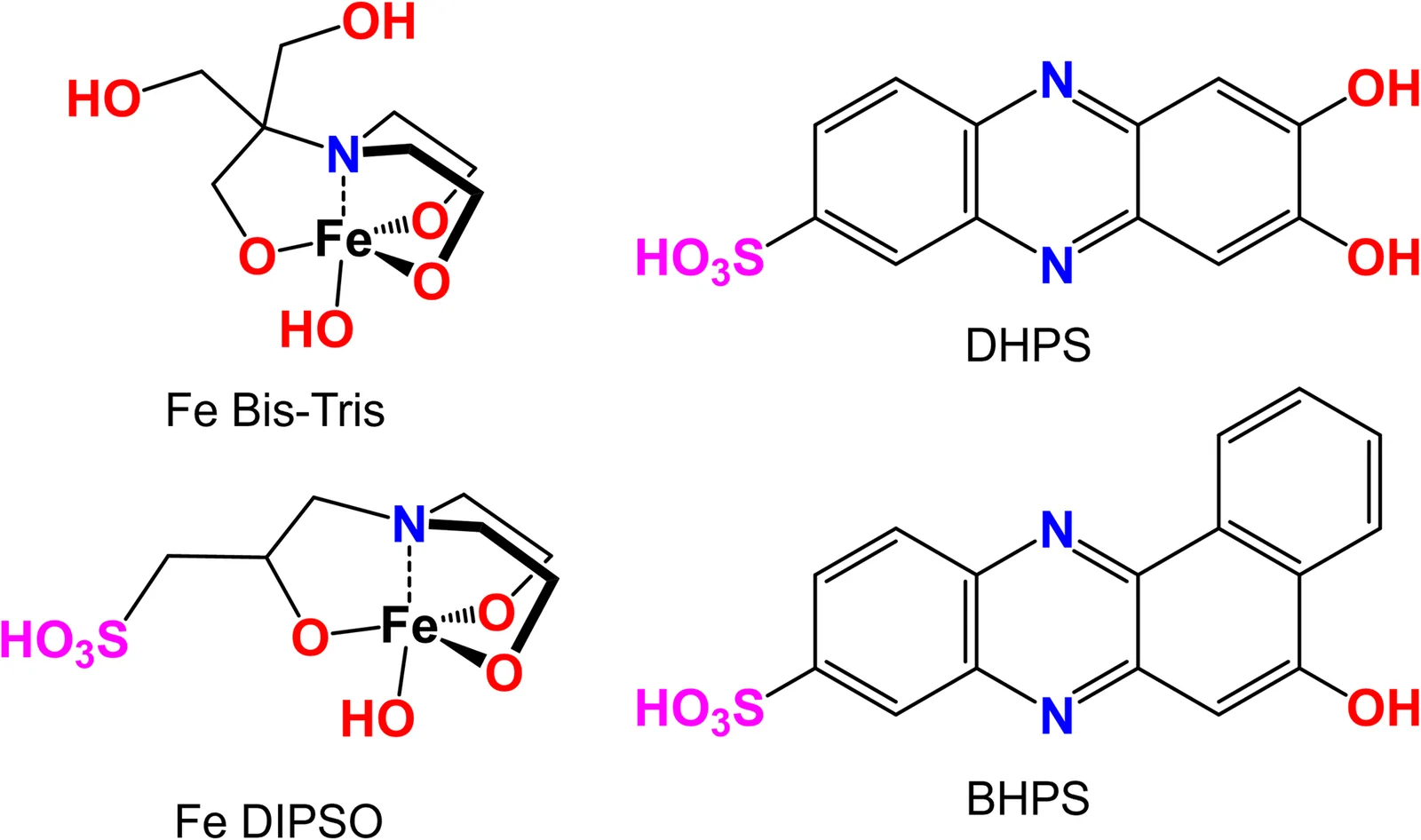
Structures of dihydroxyphenazine sulfonate (DHPS), 5-hydroxy-benzo[*a*]phenazine-sulfonic acid (BHPS), iron-2,2-bis(hydroxymethyl)-2,2′,2′-nitrilotriethanol (Fe Bis-Tris), iron-3-[bis(2-hydroxyethyl)amino]-2-hydroxypropanesulfonic acid (Fe DIPSO), the four redox mediators evaluated in this work. Each structure is labelled.

Fig. 3A shows the cyclic voltammetry (CV) of the redox mediators in 4 M NaOH with respect to the reversible hydrogen electrode (RHE). This was performed primarily to ascertain the formal potentials; however, the peak separation of the reduction and oxidation also gave an indication of the rate of charge transfer. This is crucial in enabling a high rate of hydrogen evolution, as the two possible rate limiting steps will be either (1) charge transfer from mediator to catalyst, (2) water dissociation and 
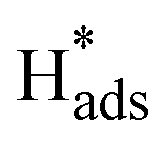
 adsorption on the catalyst surface and (3) H_2_ removal on the catalyst surface.^25^

**Fig. 3:**
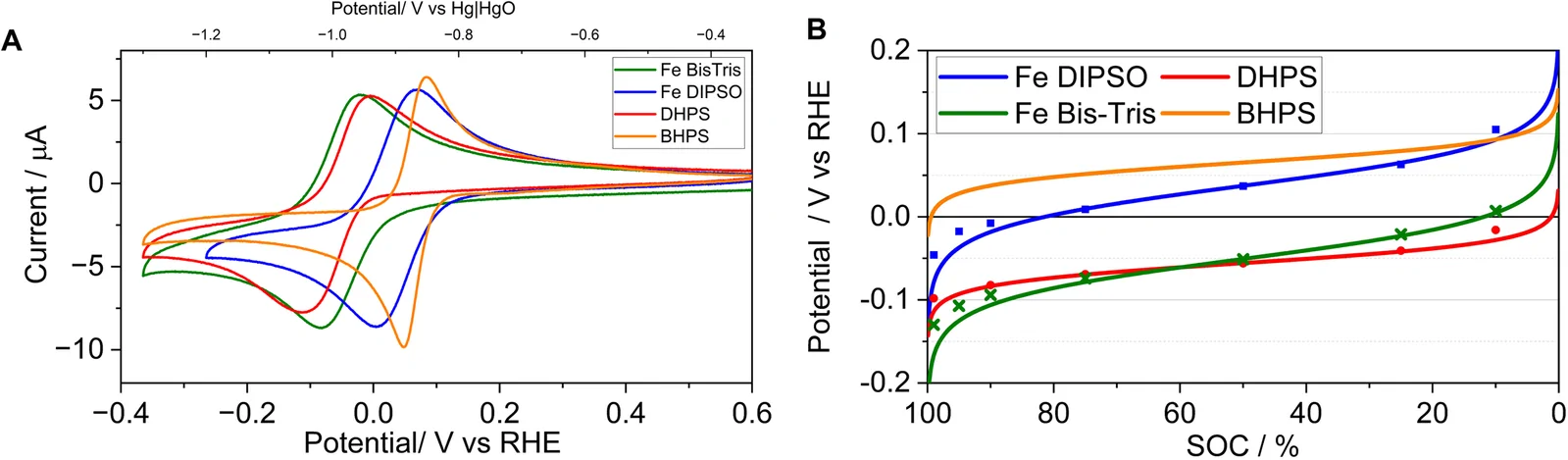
(A) Overlay of cyclic voltammograms for a selection of alkaline soluble redox mediators in 4 M NaOH. Working electrode was glassy carbon, reference electrode was Hg/HgO and the counter was a platinum wire. Scan rate is 20 mV s^−1^ and solutions were deoxygenated. (B) Overlay of the theoretical Nernst profile for the changing state of charge for each redox mediator. The redox mediators abbreviated in the figure legends are dihydroxyphenazine sulfonate (DHPS), 5-hydroxy-benzo[*a*]phenazine-sulfonic acid (BHPS), iron-2,2-bis(hydroxymethyl)-2,2′,2′-nitrilotriethanol (Fe Bis-Tris), iron-3-[bis(2-hydroxyethyl)amino]-2-hydroxypropanesulfonic acid (Fe DIPSO).

Determining the true formal potential with respect to SHE/RHE proved challenging, and as such a compromise was made between experimental and theoretical/published values for the Hg/HgO reference potential. Using an internal electrolyte with the same NaOH concentration as the analyte (4 M), we sought to eliminate the junction potential which we found had significant impact on potential measurement between our experimental reference (Hg/HgO) and our calibration references (saturated calomel and Ag/AgCl). Taking the previously reported experimental formal potentials for a range of NaOH concentrations reported in *Handbook of Reference Electrodes*, Springer 2013,^26^ we estimate a formal potential of 0.071 V *vs.* SHE for Hg/HgO in 4 M NaOH. Conversion to RHE was then made using a correction of 0.0592 V dec^−1^ and a pH of 14.6 for 4 M NaOH = 0.864 V.

In conventional electrolysers, the electrode (and thus catalyst) is held at a fixed and defined potential, whereas in the decoupled system described here, the potential experienced at the catalyst surface is defined by the redox species equilibrium quotient in the Nernst equation (eqn (1))1
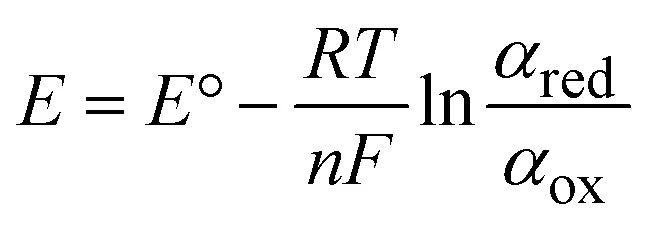
2
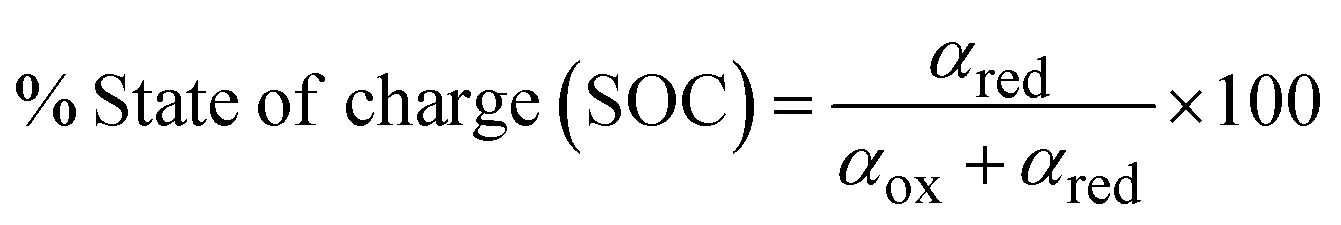


This ratio, hereafter referred to as percentage of state of charge (% SOC) (eqn (2)), can thus be used to predict the bulk potential of the solution as a measure of reaction progression. By plotting this potential as a function of % SOC, we can see how the potential experienced at the catalyst surface varies relative to the formal potential of HER across the range of mediator(s) SOC (Fig. 3B). Crucially, as the mediators have formal potentials very close to the value of 0 V *vs.* RHE (reversible hydrogen electrode, HER formal potential), it allows us to predict what portion of charge would be thermodynamically accessible for (electro)chemical discharge of the mediator to hydrogen. From Fig. 3B, we see that this results in an available charge portion of just 19% for Fe DIPSO, whereas for Fe Bis-Tris 88% of the charge has the potential for HER. DHPS, with about the same formal potential as Fe Bis-Tris, has even more charge available at essentially 100%, due to the flatter gradient resulting from it being a 2e^−^ couple. BHPS barely occupies the thermodynamically viable side of the hydrogen evolution line and would theoretically yield less than 1% hydrogen.

The solid lines shown in Fig. 3B are theoretical, however, mixtures of redox mediator at different state of charge (plotted symbols) were analysed using open circuit potentiometry and found to agree very well with the theoretical results. Some deviation was observed at the higher and lower ends of SOC, most likely because of inaccuracy in measuring the smaller of the two volumes when producing the mixture. This ability to confidently relate a measured potential to a solution SOC allows the rate of discharge of the mediator as a function of time to be determined and for the collection of kinetic data by continuous monitoring of open circuit potential (OCP). Somewhat problematically, however, was that mediators which displayed poor reversibility by cyclic voltammetry (primarily DHPS), also displayed a systematic offset from the formal potential measured by cyclic voltammetry. While symmetry of the Nernst profile remained high, the formal potential as determined from CV differed from the formal potential determined from a mixture where the ratio of reduced and oxidised mediator was 1 : 1 (predicted 10–20 mV more positive).

### Electrochemical characterisation of catalysts

Nickel materials of varying size and morphology were used as HER catalysts to compare with direct alkaline water electrolysis. While nickel foam would ideally be used in a real system to immobilise powdered catalysts, here we sought to use catalysts that could form a particulate suspension or dispersion for consistency between experiments where mediator was added to catalyst and experiments where catalyst was added to mediator by injection. As an initial benchmark, the electrochemical behaviours of a selection of nickel powders were evaluated using linear sweep voltammetry (LSV). The nickel micropowders synthesised by atmospheric plasma spraying (APS) powders were used as bought, as was the 10% platinum on Vulcan (Pt-V10). The dry Raney® nickel (R-Ni) was prepared by vacuum drying of RN2800 followed by exposure to ambient oxygen. This oxidises and reorganises the crystal structure of the metal powder but allows more rigorous weighing of the material. All powders were then prepared as inks containing a Nafion binder (0.91 mg mL^−1^ of Ni) and drop-cast onto a glassy carbon working electrode. The LSVs were obtained in a 4 M NaOH electrolyte (*ca.* pH 14.6) and are given in Fig. 4. Due to the ambiguity of defining onset potentials the performance figure of merit used was the overpotential required to achieve a current density of 10 mA cm^−2^, given in the adjacent table.^27^

**Fig. 4:**
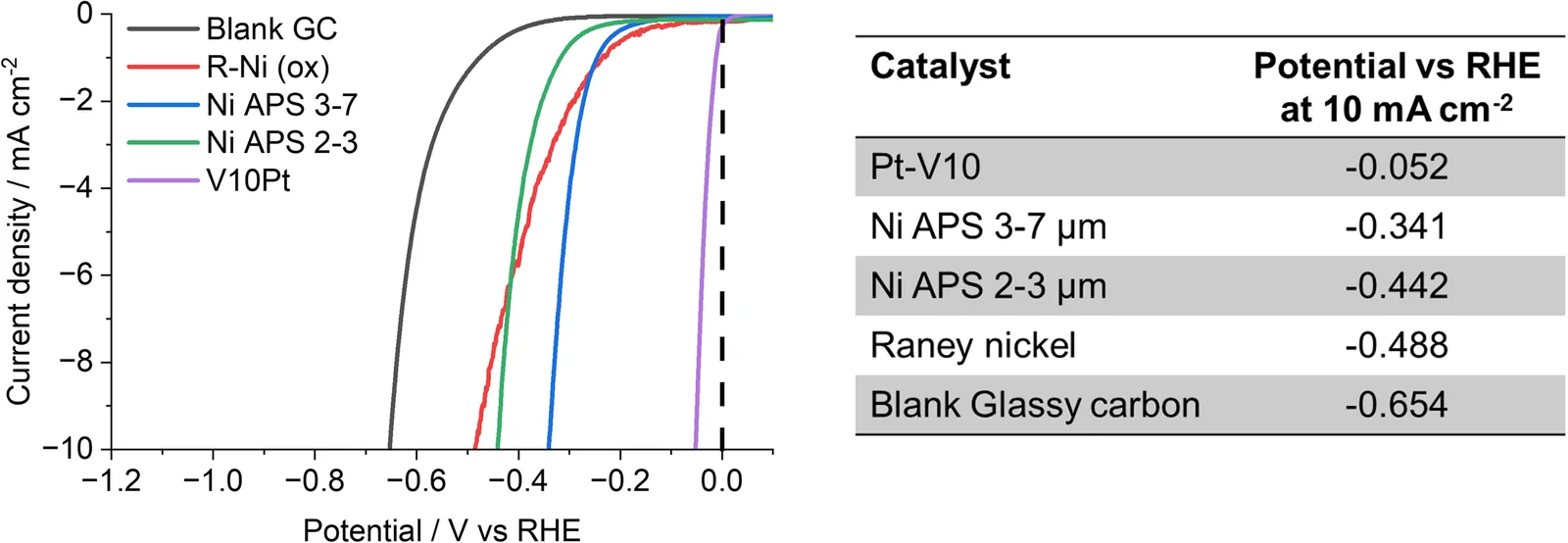
Linear sweep voltammetry of the catalyst materials dispersed on a 3 mm diameter glassy carbon electrode. Data gathered *vs.* the Hg/HgO reference electrode and corrected to the RHE in 4 M NaOH. The scan rate was 5 mV s^−1^ and solutions were deoxygenated under N_2_. Currents were normalised to zero current at 0 V RHE to improve comparison of relative performance.

The data in Fig. 4 shows similar active potentials for the nickel materials, with performance of around −0.410 V ±78 mV overpotential to achieve 10 mA cm^−2^. This corresponds to a coverage of approximately 0.64 mg cm^−2^, and therefore 15.6 mA mg^−1^ catalyst. Note that none of the data shown in Fig. 4 are *iR* compensated or corrected. It is very challenging to accurately and consistently apply *iR* correction without introducing artefacts to the data. Whilst the R-Ni has the lowest overpotential according to the LSVs for performance at 10 mA cm^−2^, it is evident from the noise in the LSV that the material is producing bubbles. R-Ni is a notoriously active HER catalyst and reportedly delivers very low overpotentials of 150 mV compared to other nickel materials. As this data is not compensated, their LSV is likely showing the effect of uncompensated resistance caused by the bubble formation within the spongy R-Ni material.^28^ The platinum catalyst performs best achieving benchmark activity at just −0.052 V *vs.* RHE, as expected. The disparity between the larger Ni APS 3–7 and the smaller Ni APS 2–3 is surprising, as one would expect the smaller, higher surface area Ni particles to improve the performance. All these LSVs were repeated and gave comparable results each time, though some variability was observed that likely resulted from material loading and the quality of the drop casting.

### Batch reactions and thermodynamic limits

Considering the redox mediators and the underpinning nickel catalyst candidates' electrochemical performance, the mediators have a redox potential more positive than the HER onset of all but the platinum catalyst. This would intuitively suggest that the mediators will not have sufficient thermodynamic driving force to effectively drive HER with the nickel catalysts. Furthermore, it is expected that there is a thermodynamic limit to the portion of charge available for HER when using the mediator as a batch reducing agent. To test this limit, a series of experiments were run pairing three of the mediators (DHPS, Fe Bis-Tris and Fe DIPSO) and catalysts in small-scale batch reactions. We elected not to use the BHPS as the potentials were insufficient for driving the HER. Within a gas tight vial, a 5 mL aliquot of >99% SOC mediator (0.1 mmol e^−^ equivalent) was added to 5 mg of catalyst. The reaction was left for >12 hours to ensure an equilibrium had been reached, and the composition of the headspace gas was analysed by gas chromatography. By considering the total volume of the headspace and the concentration of H_2_ within and dividing by the charge added by the mediator (0.1 mmol of e^−^), we can calculate an equivalent faradaic yield (FY) of the reaction (Fig. 5A), and the theoretical conversion efficiency (CE) (Fig. 5B). Based on thermodynamic estimates alone, the anticipated maximum conversions are given by the % SOC below −0 V *vs.* RHE indicated in Table 1.

**Fig. 5:**
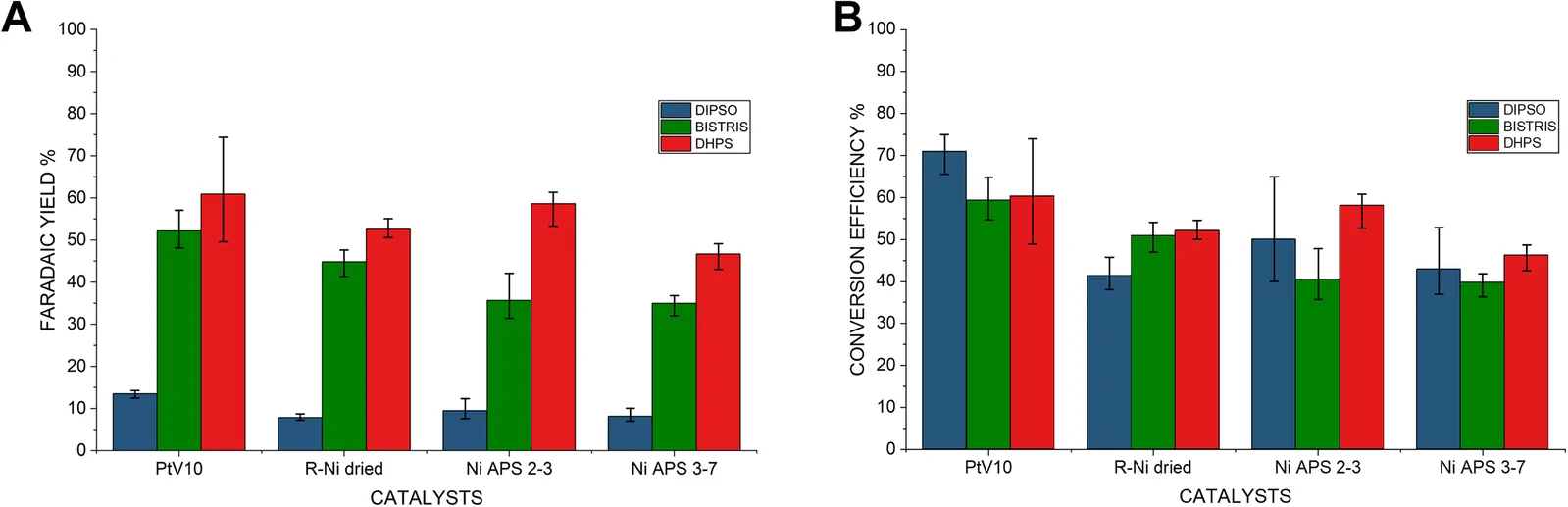
(A) Batch hydrogen faradaic yields for three mediators (Fe DIPSO, Fe Bis-Tris and DHPS) with four catalysts (Pt V10, Raney® Ni, Ni APS 2–3 and Ni APS 3–7) all in 4 M NaOH, where 100% would be the maximum charge added by the aliquot of reduced mediator. (B) The same data plotted where 100% would be the portion of charge added by the mediator that would fall below 0 V *vs.* RHE as calculated from the Nernst equation.

**Table 1:** Mediator characteristics

Mediator	*E*_f_ *vs.* HgO/RHE^a^ (V)	Peak separation (mV)	% SOC below 0 V *vs.* RHE
Fe Bis-Tris	−0.986/−0.051	62	88
Fe DIPSO	−0.898/0.037	62	19
DHPS	−0.991/−0.056	97	99
BHPS	−0.870/0.065	35	<1

a Assuming Hg/HgO reference potential of 0.071 V *vs.* SHE for an internal solution of 4 M NaOH based on potentials given in ‘*Handbook of Reference Potentials*’, Springer 2013.^26^

Fig. 5A shows that the FY varies between both mediators and catalysts. None achieve yields over 75%, yet evidently those with more driving force, the more reducing mediators (Fe Bis-Tris and DHPS), are superior. Critically, we see that mediators with formal potentials above 0 V *vs.* RHE (Fe DIPSO) are still able to drive HER to a reasonable degree with respect to what would be theoretically accessible. This is more evident in Fig. 5B, where the conversion efficiencies of each mediator are approximately equal, regardless of their respective driving forces. One possible explanation for this is that while RHE is taken with a H_2_ activity of 1, there is initially no H_2_ in the vials, which pushes the H_2_ equilibrium positive of 0 V *vs.* RHE. This is a non-equilibrium thermodynamic system whereas most thermodynamic calculations concern fully equilibrated conditions. It is nevertheless remarkable that so much of the available driving force of a species at thermodynamic potentials, is available for HER, alluding to highly efficient conversion, with a relatively consistent performance with all the catalyst materials. Despite much higher driving forces, the Fe Bis-Tris does not markedly improve conversion efficiency relative to the DIPSO except with the R-Ni catalysts, suggesting the inorganic complex itself has some impact on its interaction with the catalyst and ability to drive the reduction reaction. The phenazine, DHPS, shows a very different conversion yield, achieving 62% conversion ±12% over three separate measurements with the Pt-V10, 52% ± 2% with the dry R-Ni and 57% ± 4% with Ni APS 2–3. In general, the trends for faradaic yields are as expected with regards the redox mediator potentials (DHPS > Fe Bis-Tris > FeDIPSO) yet conversion efficiencies for each respective mediator are lower than anticipated achieving just 40–70% of this available charge. This suggests the reducing power of the redox mediators is being consumed by side processes, such as oxide reduction on the catalysts. The catalyst performances are also not reflecting their relative performances from Fig. 3. The Pt-V10 appears to underperform, suggesting that it may be compromised by the decoupled reaction and deactivated, whereas the nickel catalysts are performing better than the SVs suggest they will at these lower potentials.

### System kinetics of decoupled HER

The conversion efficiencies of the batch reactions showed nickel catalysts to be competitive to Pt in most cases. However, these batch reactions were given a full 12 hours to complete and appeared very sluggish, with the exception of the Pt catalyst tests which observed immediate visual changes in mediators. To further understand the differences in observed behaviour between the conventional three-electrode voltammetry and the batch deHER reactions for the various catalysts, a series of kinetic experiments were performed using Pt-V10 and the best Ni catalyst, R-Ni, both using the best mediator, DHPS, as shown in Fig. 6. A combination of chronopotentiometry, monitoring the OCP of the solution with respect to a known reference as a measure of SOC with respect to time, and gas chromatography (GC) at regular intervals, monitoring the production of H_2_ over time, was employed. Initial GC samples were taken in quick succession, followed by measurements every 5 minutes. While essentially continuous, OCP measurement appeared to be highly susceptible to reference electrode drift over these time frames, resulting in a high possibility for error and therefore lower confidence in % SOC measurements.

**Fig. 6:**
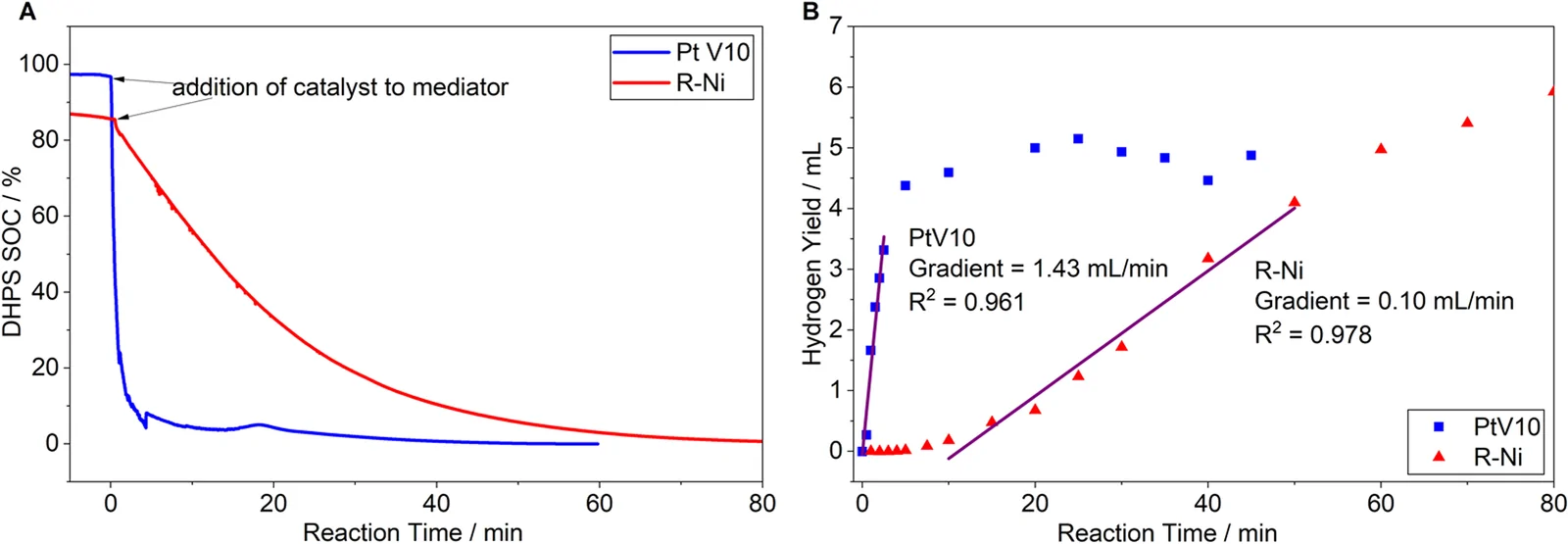
Evaluation of reaction kinetics of DHPS discharge using Pt-V10 and R-Ni catalysts. 50 mg of each catalyst used with 10 mL of 0.1 M DHPS. (A) Overlay of the OCCP measurement using a glassy carbon electrode and Ag/AgCl leakless reference electrode for each catalyst. (B) Head space hydrogen quantities with respect to time measured using GC-TCD.

A comparison of the initial rates between Pt-V10 and R-Ni catalysts determined that the Pt catalyst is much more immediately active than the R-Ni. The initial rate for Pt-V10 of 1.43 mL H_2_ min^−1^ was ten times higher than for R-Ni (0.10 mL H_2_ min^−1^), with hydrogen immediately measurable upon addition of catalyst to DHPS. Indeed, when a loading of 50 mg was used the reaction was essentially complete in the first 5 minutes. This is compared to the R-Ni which displayed a considerable lag time of 5 minutes before any HER was detected at a quantifiable level. However, on starting to be catalytically active the nickel appeared to convert more equivalents of the DHPS to hydrogen, achieving *ca.* 60–70% conversion compared to *ca.* 40% of the Pt-V10.

The lag in the commencement of HER on the R-Ni suggests there is a pre-reaction that is consuming the charge. The R-Ni discharge profile exhibits two apparent kinetic regimes. An initial near-linear loss of mediator charge occurs before detectable hydrogen production, suggesting consumption of reducing equivalents during catalyst activation. Following the onset of hydrogen evolution, the SOC decay becomes approximately exponential, consistent with mediator-dependent catalytic turnover, though not first order. This was a consistent profile over a number of measurements using 50 mg and 500 mg of R-Ni catalyst. It is possible that the catalyst is coated in an oxide due to the drying procedures undertaken to eliminate water from the suspension reaction. The reduced redox mediator is evidently reacting with the R-Ni to reduce the catalyst surface, before a point is reached where it becomes activated towards HER. Thereafter, a steady consumption of charge continues to be monitored by OCP in line with the generation of hydrogen. This parasitic reduction reaction may be the reason for the lower faradaic yield, as electrons are consumed as oxide reductant rather than used in proton reduction. Experiments exploring the reuse of the catalysts will be needed to elucidate this mechanism further.

A tentative measure of the apparent initial rates for Pt-V10 gives 26.6 mL min^−1^ g^−1^ for 50 mg loading, while for R-Ni the initial rate was 1.59 mL min^−1^ g^−1^. However, due to the large variance in the final H_2_ yield between experiments, it is impossible to determine if this observed effect of loading is real. Indeed, we would not expect mass activity to increase as loading is increased for Pt-V10 if the reaction was limited by mass transport, as this would be expected to hinder the reaction when operating at a higher overall rate. Nor would we expect the mass activity of R-Ni to change with respect to loading if the loading were the primary limit to the rate of reaction.

The most significant observation throughout the experiments was the very poor rate per catalyst loading for the nickel materials in the highly alkaline medium, compared to that previously reported for acidic,^11,29^ and near-neutral conditions.^7,20^ The high activity of the near-neutral example can be explained primarily by the excessively large driving force provided by the Cr(ii) mediator used therein (*E*° = −0.6 V *vs.* RHE, 1 M KHCO_3_ electrolyte). However, the V(ii) mediator used by Girault *et al.* provided much less driving force at only *E*° = −0.26 V *vs.* RHE (1 M H_2_SO_4_ electrolyte),^11^ although this is still much more overpotential that any of the mediators tested here at pH 14.6 are able to provide. The poor rate of reaction meant that it was feasible to employ headspace hydrogen analysis during the reaction and acquire a proxy for hydrogen production rates.

HER in alkaline conditions is a more kinetically challenging reaction, with two-orders of magnitude lower rates than in acidic conditions.^30^ Water reduction is considered in three possible stages, Volmer, Heyrovsky and Tafel, which in alkaline conditions are as follows (eqn (3)–(5));3
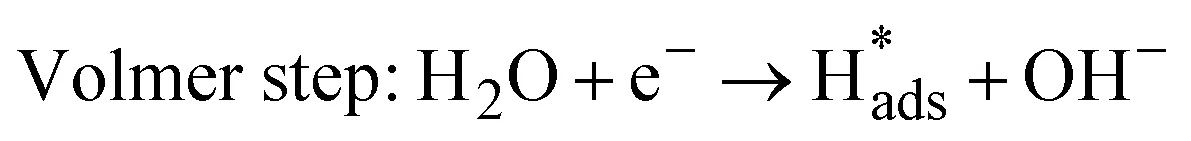
4
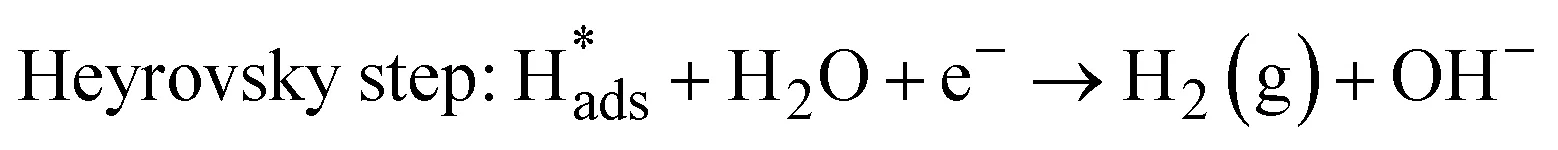
5
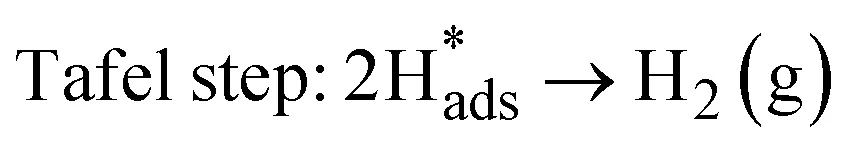
These elementary reaction steps are a challenge to understand and define in conventional alkaline electrolysers, yet decoupling the reaction to relate redox mediator equilibration to the Fermi level of dispersed catalyst material adds further complexity. This is simply described in Fig. 7. To date, it is generally accepted that alkaline HER catalysts are best if they can facilitate the initial dissociation of the water molecules, thus requiring oxophilic materials.^30^ While we would expect the HER to occur by the same mechanism as in conventional electrolysers from the perspective of the catalyst, that is the Volmer step of H* adsorption and either the Tafel or Heyrovsky step of H_2_ formation and desorption, the initial step of electron transfer from the mediator to the catalyst could also have several effects on the overall kinetics. The first to consider is the charge transfer kinetics of the mediator. A slow rate of charge transfer from mediator to catalyst (*k*_1_) may severely limit the reaction, as this would affect the potential of the highest energy electrons on the catalyst. If electrons are consumed quickly by H_2_ adsorption and formation (*k*_2_) but only transfer slowly from the mediator, then the Fermi level of the catalyst particle will be the potential of HER at that rate. Meanwhile, if electron transfer is fast and H_2_ formation is slow, the Fermi level of the catalyst particle will be the potential of the mediator solution. If the rates are competitive, then the Fermi level of the catalyst will be at an intermediate potential between these two processes.

**Fig. 7:**
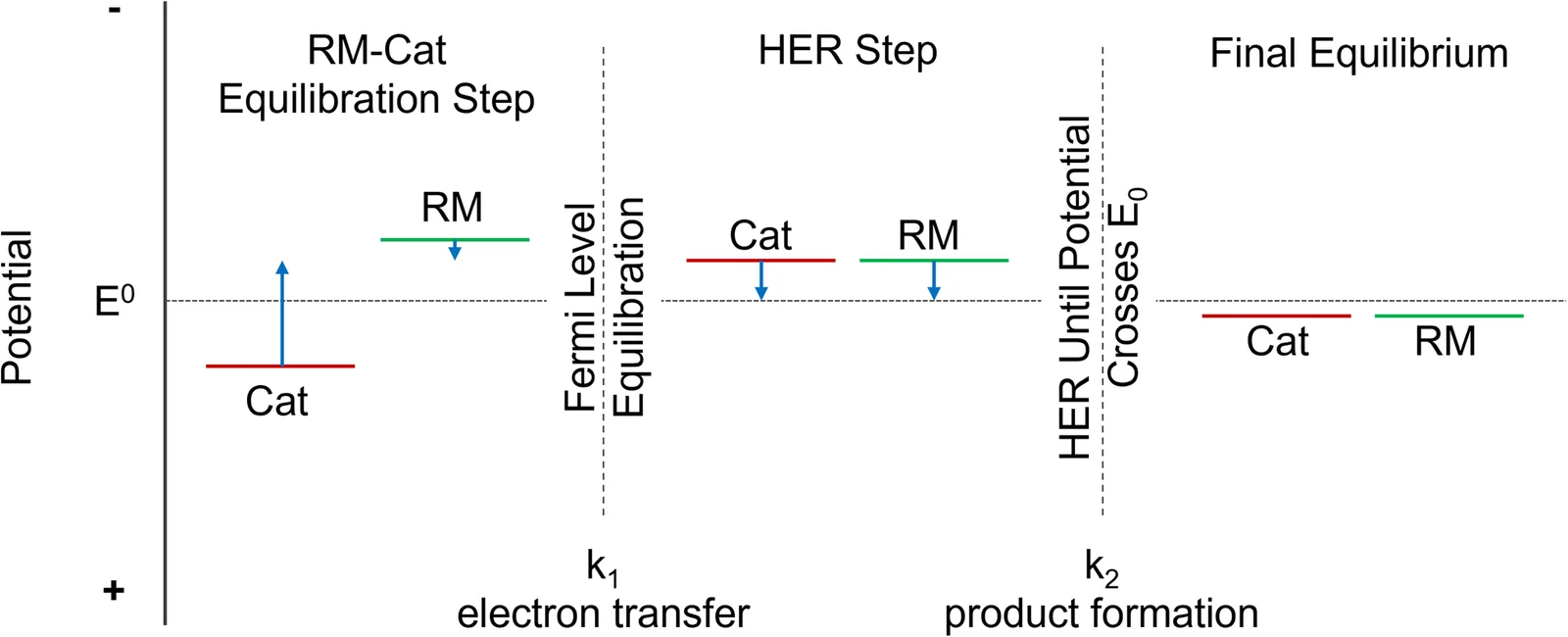
Schematic of the equilibration between the catalyst Fermi levels and the solution potential.

A further effect that needs to be considered is that of the proton coupled mediators. When these mediators transfer their charge to the catalyst, they also release protons directly adjacent to the catalyst surface, lowering the local pH. This will in turn result in a positive shift of the RHE, which will increase the overpotential provided for the reaction and conceivably increase its rate relative to non-proton-coupled mediators. When considering the paired oxidation however, it is still important to choose a posolyte that is proton coupled so that the battery can remain pH balanced. The detailed study by Zhang *et al.*^17^ proposed that HER mediated by DHPS on platinum catalysts occurs by a concerted proton–electron transfer, where atomic hydrogen is transferred directly onto the catalyst surface, bypassing the unfavourable initial water dissociation step at high pH. They predicted a lower energy barrier to this process compared to direct water dissociation, effectively lowering the overpotential requirement for HER.

The equilibration of potential between the mediator and the catalyst can be considered equivalent to the process which occurs at the electrode surface during conventional electrochemistry of a redox active species. At the instant of polarisation, a net charge transfer gradient is immediately established, in this case a flow of electrons from the mediator to the catalyst particle. As the Fermi level of the catalyst increases, that is the minimum energy of the highest energy electrons, the possibility of charge transfer back to the mediator increases. Eventually, an equilibrium is established, where the rate of electron transfer from mediator to catalyst and *vice versa* are equal (eqn (6)). This is the fundamental reason why the potential of the solution changes as a function of SOC, because as one redox state of the mediator becomes more active than the other, the equilibrium rate of the two directions of electron transfer will stabilise at different potentials.6
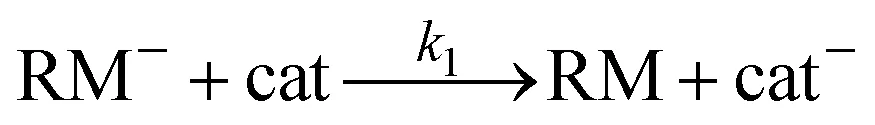
If electrons are being consumed on the catalyst surface for processes other than charge transfer to the mediator, such as the effectively irreversible HER (as shown by eqn (7)), this will compete with the reverse transfer, altering the potential of the catalyst surface relative to the mediator. Indeed, a steady state will develop in which the rate of electron flux from mediator to catalyst is the same as the rate of H_2_ production by the catalyst, as given by the overall reaction in eqn (8).7
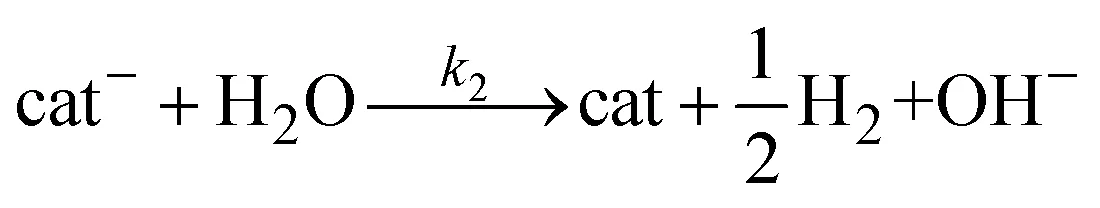
8
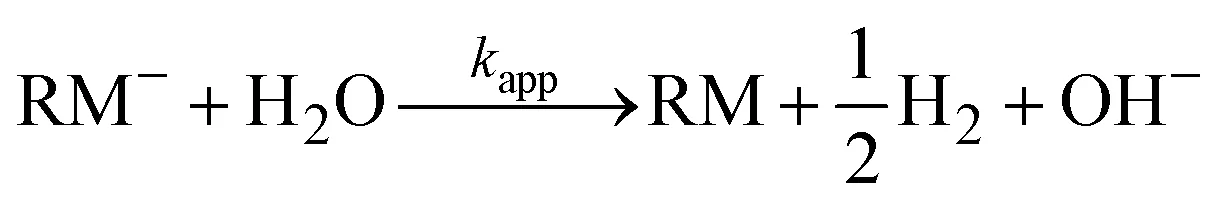
This however does not consider the possibility of an energy barrier to charge transfer, such as surface or molecule restructuring which could severely limit the rate of electron transfer in one or both directions. Of course, under catalytic production of H_2_, no true equilibrium exists, making it difficult to measure the true potential of the catalyst particles during the reaction.

## Conclusions

In this work, we have investigated redox-mediated hydrogen evolution in strongly alkaline media using nickel-based catalysts and redox mediators with only moderate thermodynamic driving force. The results demonstrate that decoupled electrolysis operates within a fundamentally different kinetic regime from conventional electrocatalysis. By separating electrochemical charge storage from catalytic hydrogen evolution, the kinetic penalty associated with the electrode/electrolyte interface is largely circumvented, enabling efficient hydrogen production at potentials approaching the thermodynamic limit, even with conventional nickel catalysts.

Despite the absence of direct electrode-mediated electron transfer, catalyst identity remains critical in determining reaction kinetics. Comparison of Pt-V10 and Raney® nickel reveals markedly different catalytic behaviour: platinum rapidly converts the reduced mediator to hydrogen within minutes, whereas Raney® nickel exhibits a pronounced induction period before sustained hydrogen evolution is established. These observations indicate that while interfacial charge-transfer limitations are alleviated, the intrinsic surface chemistry of the catalyst continues to govern the overall reaction rate.

We propose that these observations reflect a distinct mechanistic regime in which the catalyst equilibrates electronically with the dissolved redox mediator, transiently storing charge before gradually driving hydrogen evolution according to its intrinsic surface kinetics. Rather than continuous electron injection from an electrode, catalytic turnover is sustained by chemical electron transfer from the mediator, fundamentally changing how electrons are delivered to catalytic sites.

From a practical perspective, coupling this chemistry with flow-battery architecture offers the opportunity to reduce mediators electrochemically under conditions that are more efficient than direct hydrogen evolution on nickel electrodes, while subsequently generating hydrogen chemically at inexpensive catalysts. Spatial and temporal decoupling therefore reframes the conventional relationship between electrochemical kinetics and catalyst performance, offering a route to reduced precious-metal dependence, improved electrochemical efficiency, and greater operational flexibility.

More broadly, these findings establish decoupled electrolysis as a distinct model system for probing the interplay between electron transfer and heterogeneous catalysis. By disentangling interfacial charge transfer from catalytic turnover, this approach provides new mechanistic insight into electrocatalytic hydrogen evolution and opens opportunities for the rational design of future redox-mediated energy conversion systems.

## Author contributions

M. P., L. P., and K. E. T. conceptualised the project. L. P. and M. P. synthesised the materials. L. P. and M. P. performed the experiments and analysed the results, with assistance from K. E. T. All authors wrote the manuscript and contributed to revision. K. E. T. provided supervision and obtained funding.

## Conflicts of interest

There are no conflicts to declare.

## Supplementary Material

FD-OLF-D6FD00117C-s001

## Data Availability

Data for this article, including raw electrochemical data, gas chromatography and data processing files, are available at Zenodo at https://doi.org/10.5281/zenodo.21645133. Supplementary information (SI): additional calculations and calibrations data. See DOI: https://doi.org/10.1039/d6fd00117c.

## References

[cit1] Ifkovits Z. P., Evans J. M., Meier M. C., Papadantonakis K. M., Lewis N. S. (2021). Energy Environ. Sci..

[cit2] Li Z., Sun L., Zhang Y., Han Y., Zhuang W., Tian L., Tan W. (2024). Coord. Chem. Rev..

[cit3] Wallace A. G., Symes M. D. (2018). Joule.

[cit4] McHugh P. J., Stergiou A. D., Symes M. D. (2020). Adv. Energy Mater..

[cit5] Paul A., Symes M. D. (2021). Curr. Opin. Green Sustainable Chem..

[cit6] Potter M., Toghill K. E. (2026). Dalton Trans..

[cit7] Potter M., Smith D. E., Armstrong C. G., Toghill K. E. (2024). EES Catal..

[cit8] Stergiou A. D., Broadhurst D. H., Symes M. D. (2023). ACS Org. Inorg. Au.

[cit9] Huang Y., Zhou H., Wang J., Zhang J., Guan J., Wu R., Cheng F., Zhang B. (2026). Nat. Commun..

[cit10] Wang F., Li W., Wang R., Guo T., Sheng H., Fu H.-C., Stahl S. S., Jin S. (2021). Joule.

[cit11] Amstutz V., Toghill K. E., Powlesland F., Vrubel H., Comninellis C., Hu X., Girault H. H. (2014). Energy Environ. Sci..

[cit12] Reynard D., Girault H. (2021). Cell Rep. Phys. Sci..

[cit13] Piwek J., Dennison C. R., Frackowiak E., Girault H., Battistel A. (2019). J. Power Sources.

[cit14] Symes M. D., Cronin L. (2013). Nat. Chem..

[cit15] Clyde Hydrogen, https://clydehydrogen.com/, accessed 28 May 2026

[cit16] Jin B., Wang H., Ji Y., Cheng J. (2026). J. Mater. Chem. A.

[cit17] Zhang F., Zhang H., Salla M., Qin N., Gao M., Ji Y., Huang S., Wu S., Zhang R., Lu Z., Wang Q. (2021). J. Am. Chem. Soc..

[cit18] Shao W., Chen S., Zhao H., He Y., Lu B., Shao W., Li J., Zhao Y., Wei X., Zhang C., Zhang F. (2025). J. Colloid Interface Sci..

[cit19] Ji Y., Zhang F., Zhou M., Yu J., Wang Q. (2020). Int. J. Hydrogen Energy.

[cit20] Pinho L., Potter M., Toghill K. E. (2026). J. Mater. Chem. A.

[cit21] Hollas A., Wei X., Murugesan V., Nie Z., Li B., Reed D., Liu J., Sprenkle V., Wang W. (2018). Nat. Energy.

[cit22] Park J., Lee Y., Yun D., Kim D., Hwang G., Han B., Kim Y., Jung J., Jeon J. (2023). Electrochim. Acta.

[cit23] Shin M., Noh C., Kwon Y. (2023). Chem. Eng. J..

[cit24] Shin M., Noh C., Chung Y., Kwon Y. (2020). Chem. Eng. J..

[cit25] Reynard D., Bolik-Coulon G., Maye S., Girault H. H. (2021). Chem. Eng. J..

[cit26] Handbook of Reference Electrodes, ed. G. Inzelt, A. Lewenstam and F. Scholz, Springer, 2013

[cit27] An H., Park W., Shin H., Chung D. Y. (2024). EcoMat.

[cit28] Son H., Lee H., Lee J., Han T., Lee H., Park C.-S., Jung K., Kim J., Jung H. W., Kang K.-S. (2026). J. Power Sources.

[cit29] Peljo P., Vrubel H., Amstutz V., Pandard J., Morgado J., Santasalo-Aarnio A., Lloyd D., Gumy F., Dennison C. R., Toghill K. E., Girault H. H. (2016). Green Chem..

[cit30] Baek D. S., Lee J., Lim J. S., Joo S. H. (2021). Mater. Chem. Front..

