# Supplementary material for: Electrolysis without electrodes: thermodynamic and kinetic insights into decoupled alkaline water electrolysis
Source: Faraday Discuss. 2026 Aug 4. Online ahead of print. doi: 10.1039/d6fd00117c (PMC13657250; doi:10.1039/d6fd00117c)
Supplement: Supplementary file 1 [file FD-OLF-D6FD00117C-s001.pdf]

Supplementary Information (SI) for Faraday Discussions.
This journal is © The Royal Society of Chemistry 2026

Supplementary Information

Electrolysis Without Electrodes: Thermodynamic and Kinetic Insights into Decoupled Alkaline Water Electrolysis

Mark Potter, Luís Pinho, Kathryn E. Toghill*

Department of Chemistry, Lancaster University, Lancaster, LA1 4YB, United Kingdom

*Corresponding Author: K.E. Toghill k.toghill@lancaster.ac.uk

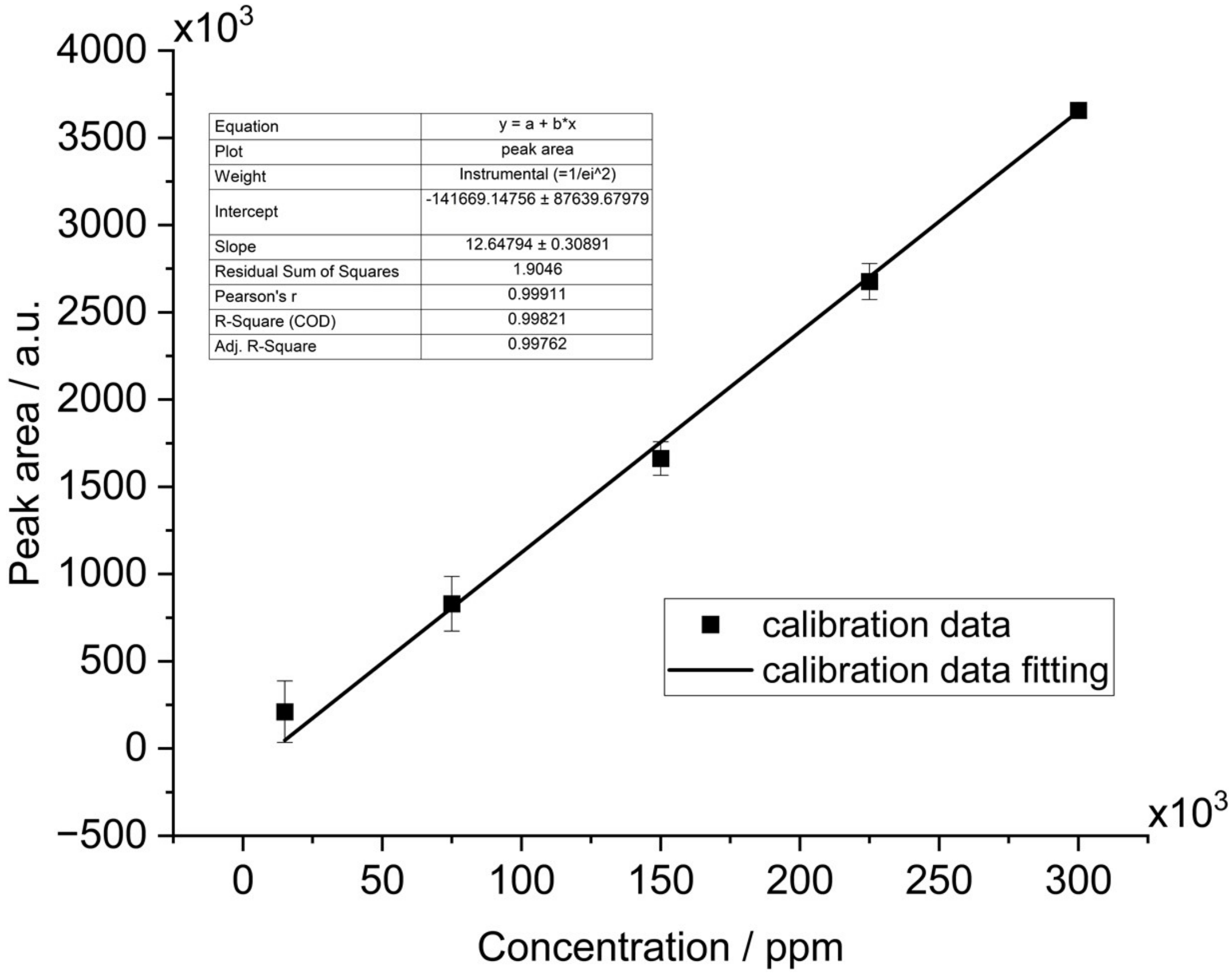

Figure S1. Gas chromatography calibration for hydrogen concentration. Samples were prepared from a calibration standard containing 300000 ppm H_2 with N_2 balance. Dilutions were performed in a glass syringe setup in N_2 to give concentrations of approximately 225000, 150000, 75000 and 15000 ppm. The calibration range has strong correlation, allowing for high confidence in the accuracy of determined concentrations.

Equations in the form y = mx + c were used to determine the concentration of H_2 in a real sample from the GC peak area;

Peak area = (12.167 × ppm) - 53123

Gas quantification and product analysis

To determine the faradaic yield for H_2, headspace gas was taken from the flask and analysed by gas chromatography. An aliquot of gas was collected in a gas-tight lock-in syringe. The sample

was then directly injected into the GC inlet for separation. Using the above calibration plot, the concentration of H_2 was determined in ppm (molar ratio). It is assumed that 100% of all gaseous products were in the headspace, as H_2 is sparingly soluble in water.

The amount of H_2 in moles within the headspace was determined as;

$$\text{Moles H2 } [mol] = \frac{\text{Headspace concentration [ppm]}}{10^6} \times \text{Total moles of gas in headspace [mol]}$$

where the ideal gas law was used to determine the total number of moles within the headspace;

$$\text{Total moles of gas in headspace} = \frac{\text{Headspace pressure } [Pa] \times \text{Headspace volume } [m^3]}{\text{Ideal gas constant } [J\,K^{-1}\,mol^{-1}] \times \text{Temperature [K]}}$$

By considering the electron stoichiometry, the amount of charge needed to form each product can be calculated;

$$\text{Charge required } [C] = \text{Moles H2 } [mol] \times \text{Electron stoichiometry} \times \text{Faraday constant } [C\,mol^{-1}]$$

which can then be compared to the total charge passed;

$$\text{Charge passed [C]} = \text{Concentration of mediator } [M] \times \text{Volume of mediator [L]} \times \text{Electron stoichiometry} \times \text{Faraday constant } [C\,mol^{-1}]$$

to determine the faradaic yield as a quantum yield;

$$\text{Faradaic yield} = \frac{\text{Charge required } [C]}{\text{Charge passed } [C]} \times 100$$

Electrochemical set-up

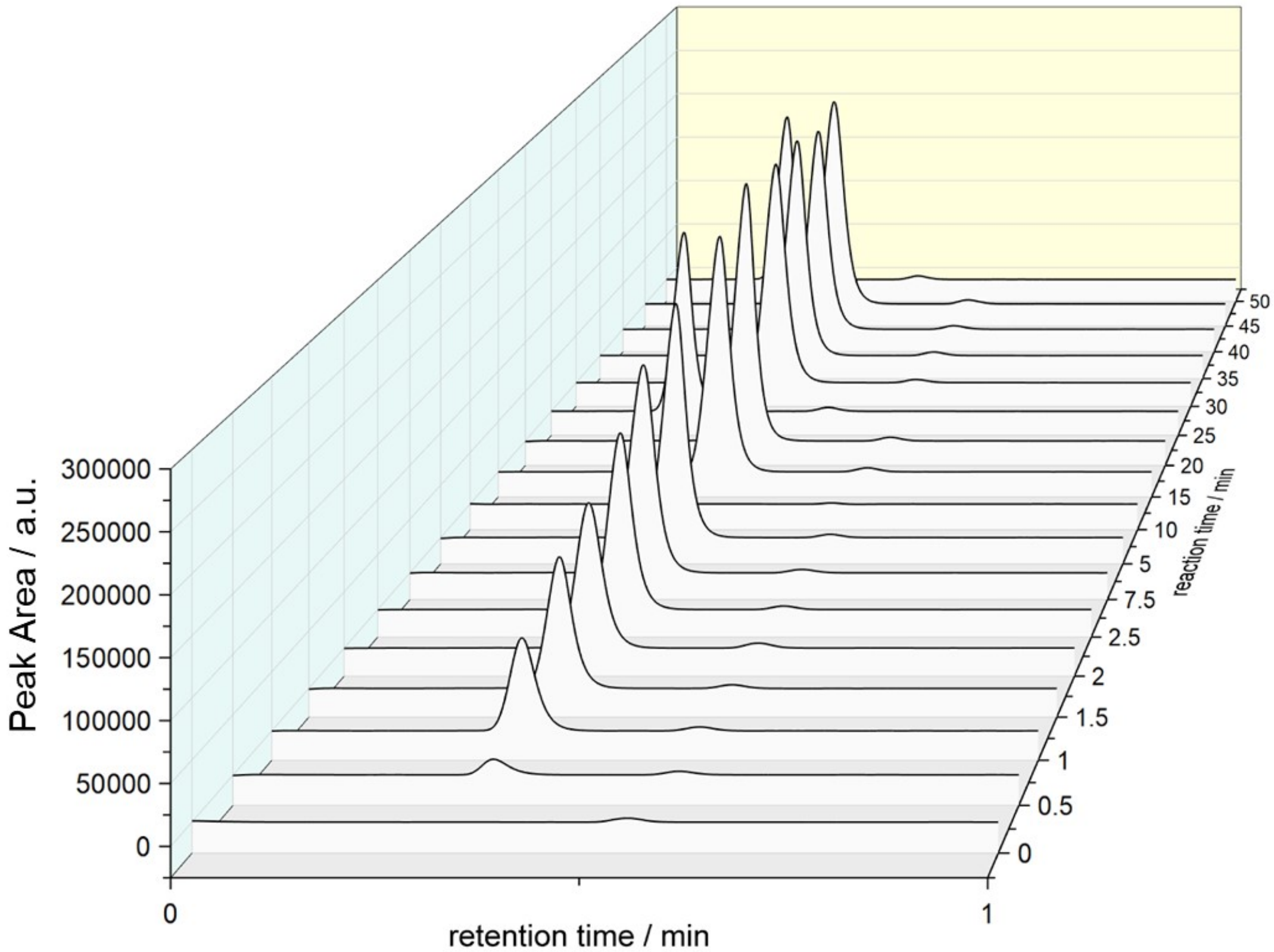

Figure S2. Representative kinetic GC traces for V10 50 mg. H_2 has a retention time of ~0.8 min

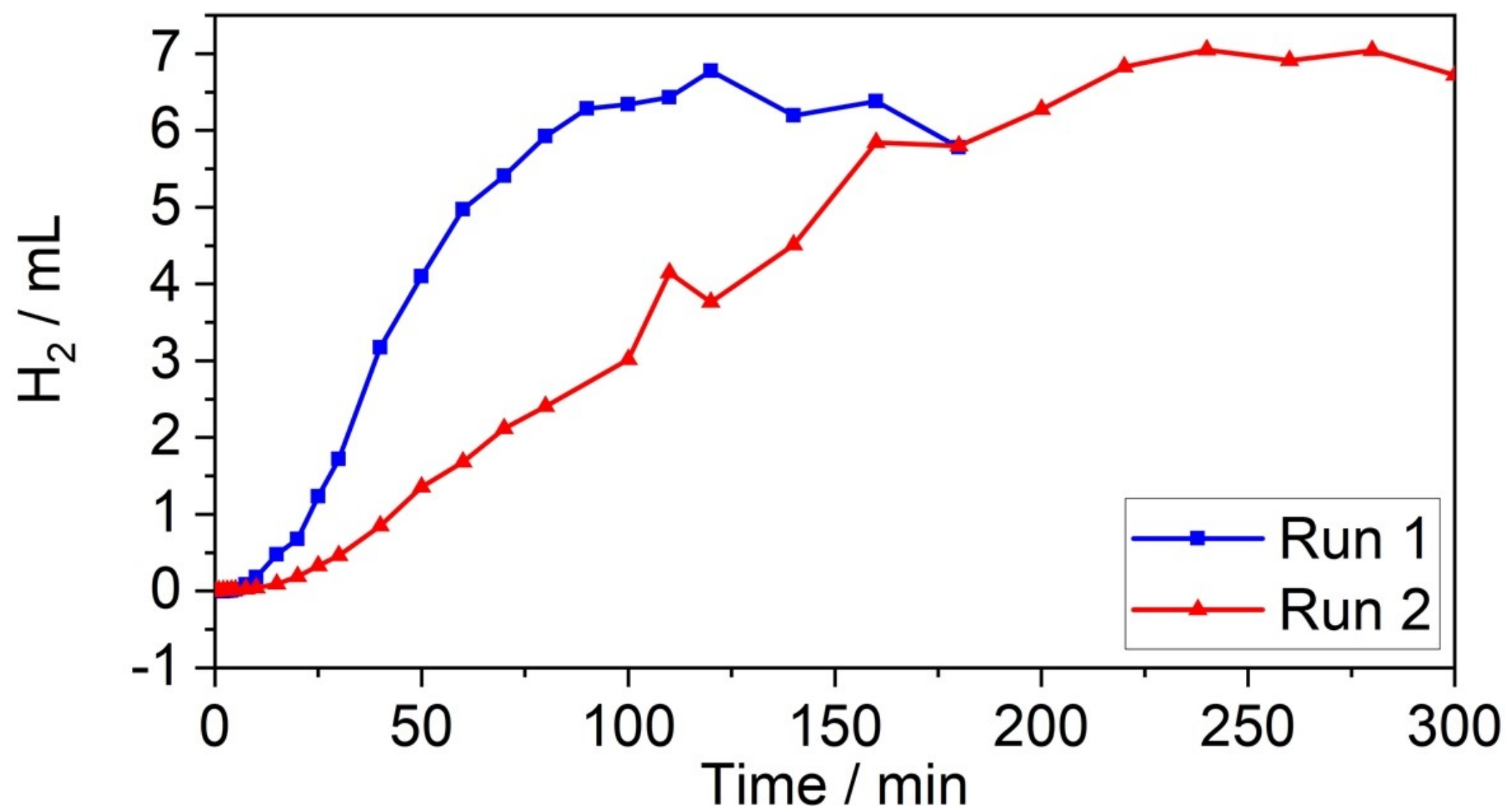

Figure S3. Cumulative H_2 volume produced as a function of time for 50 mL of 10 mM DHPS discharged by 50 mg of R-Ni catalyst.

Conversion of OCP values to SOC was performed by reversing the Nernst equation to give the reaction quotient as the subject:

$$Q_r = \frac{\alpha_{red}}{\alpha_{ox}} = e^{\frac{(E_0 - E)zF}{RT}}$$

Where E_0 is the formal potential of the redox mediator and E is the measured potential of the solution (both against the same ref), R is the ideal gas constant, T is temperature, F is the faraday constant, and z is the electron stoichiometry.

This is then normalized to state of charge for a given concentration by:

$$\%State\ of\ charge\ (SOC) = \frac{\alpha_{red}}{\alpha_{ox} + \alpha_{red}} \times 100$$

By this method, a plot of potential vs time can be converted to a plot of SOC vs time.

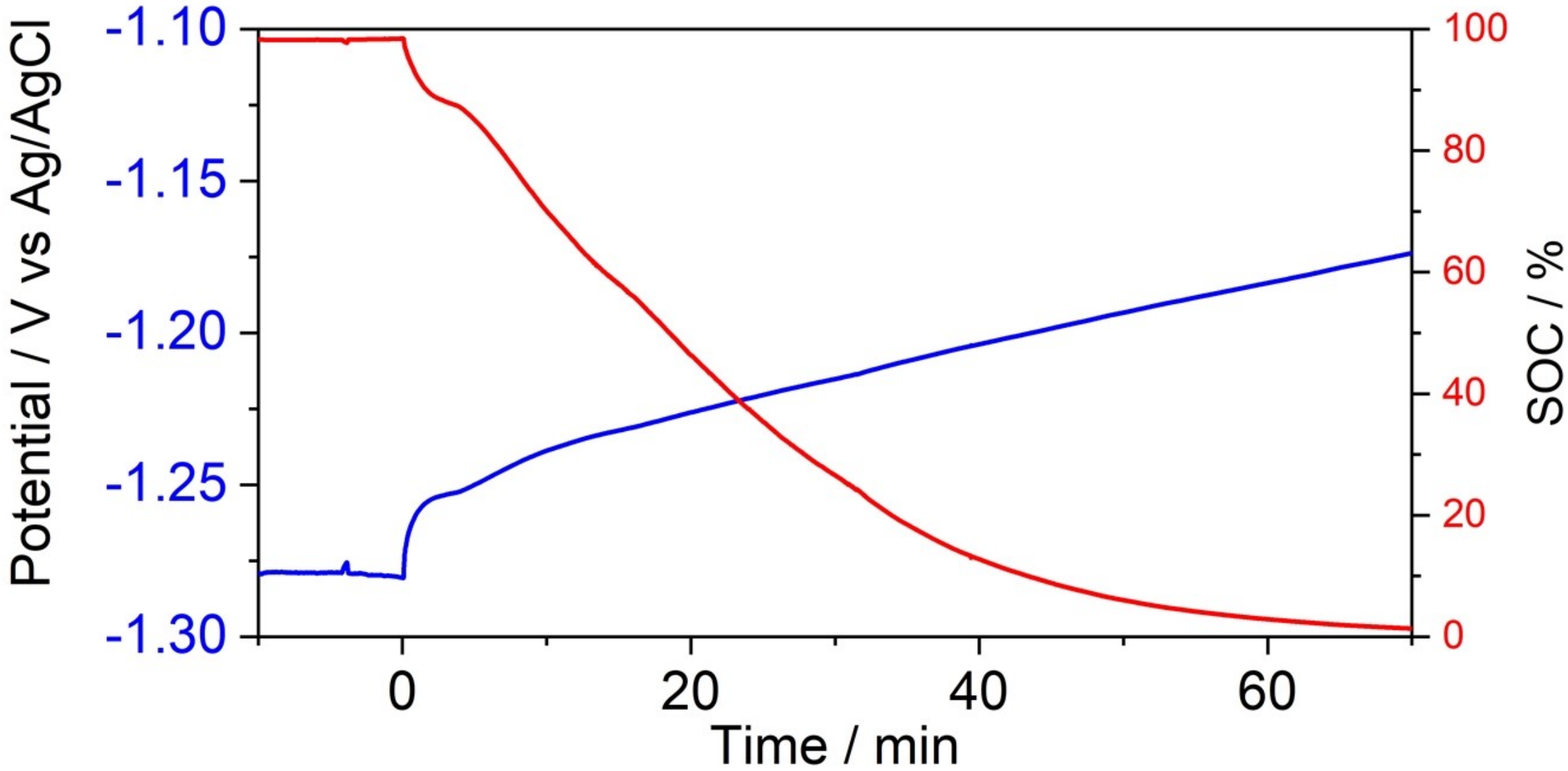

Figure S4. Graph of potential vs time (blue, left axis) and the respective conversion to SOC vs time (red, right axis) for 50 mL of 10 mM DHPS discharged by 500 mg of R-Ni catalyst, where E_0 was measured to be -1.228 V from the OCP of a 50% SOC solution.

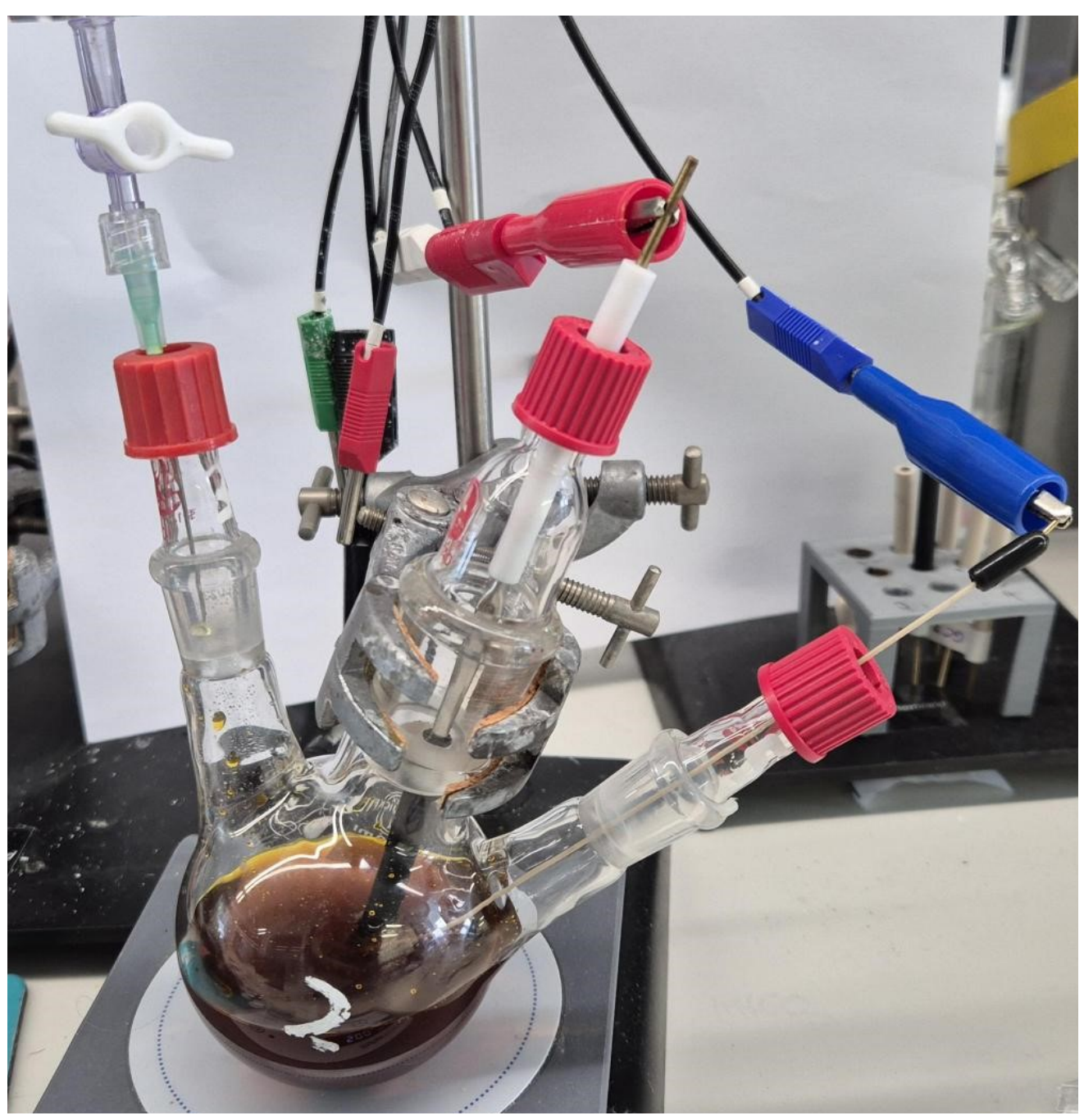

Figure S5. Round bottom flask used as reaction vessel for kinetic experiments. The solution potential was measured on a glassy carbon electrode (red clip), referenced against a leakless AgCl reference electrode (blue clip) (innovative instruments™). Gas samples were taken from the headspace via a needle and stop tap to avoid repeatedly puncturing the septum. The system was flushed with N_2 prior to injection of the charged mediator solution. The initial SOC was measured

for some time, after which the catalyst was injected, suspended in 5 mL of 4 M NaOH saturated with N_2.
